# FVC, but not FEV_1,_ is associated with clinical outcomes of asthma-COPD overlap

**DOI:** 10.1038/s41598-022-15612-w

**Published:** 2022-08-15

**Authors:** Tai Joon An, Chin Kook Rhee, Yong Bum Park, Kwang-Ha Yoo, Hyoung Kyu Yoon

**Affiliations:** 1 Division of Pulmonary and Critical Care Medicine, Department of Internal Medicine, Yeouido St. Mary’s Hospital, College of Medicine, The Catholic University of Korea, 63 ro 10, Yeongdeungpo-gu, Seoul, 07345 Republic of Korea; 2 Division of Pulmonary and Critical Care Medicine, Department of Internal Medicine, Seoul St. Mary’s Hospital, College of Medicine, The Catholic University of Korea, Seoul, Republic of Korea; 3 Division of Pulmonary, Allergy, and Critical Care Medicine, Department of Internal Medicine, Hallym University Kangdong Sacred Heart Hospital, Seoul, Republic of Korea; 4 Division of Pulmonary, Allergy, and Critical Care Medicine, Department of Internal Medicine, Konkuk University Hospital, School of Medicine, Konkuk University, Seoul, Republic of Korea

**Keywords:** Asthma, Chronic obstructive pulmonary disease

## Abstract

The effects of forced vital capacity (FVC) on clinical outcomes of asthma-chronic obstructive pulmonary diseases overlap (ACO) are still unknown. We conducted this study to examine the association of FVC on clinical outcomes in ACO. Data from the Korean COPD Subgroup Study cohort were analyzed. Patients who fulfilled the ACO criteria were included and grouped according to FVC changes, such as FVC-incline and FVC-decline. No significant differences were observed between the FVC-incline and FVC-decline groups in baseline clinical characteristics. In a year after, FVC-decline group experienced more moderate (47.1% vs. 36.8%, *p* = 0.02) and moderate-to-severe (49.8% vs. 39.6%, *p* = 0.03) acute exacerbations (AEs), compared to FVC-incline group. The frequency of moderate AEs (1.3 ± 2.1 vs. 0.9 ± 1.7, *p* = 0.03) and moderate-to-severe AEs (1.5 ± 2.5 vs. 1.1 ± 1.9, *p* = 0.04) were higher in the FVC-decline group than in the FVC-incline groups. After adjusting for confounding factors, FVC-decline group was associated with moderate AEs (odds ratio [OR] = 1.58; 95% confidence interval [CI] 1.02–2.44; *p* = 0.04), and moderate-to-severe AEs (OR = 1.56; 95% CI 1.01–2.41; *p* < 0.05) in ACO patients, which was not seen in FEV_1_ changes. FVC changes are associated with clinical outcomes in ACO.

## Introduction

Asthma and chronic obstructive pulmonary disease (COPD) are chronic inflammatory airway diseases that are common and burdensome worldwide^1–3^. Features of asthma and COPD can co-exist in the same patient, which is called asthma-COPD overlap (ACO)^4–6^. Asthmatic features in ACO patients include high blood eosinophil and immunoglobulin E levels, a large post-bronchodilator (BD) response, and a history of asthma, atopy, or allergic rhinitis. COPD features in ACO are associated with exposure to smoking or air pollution, typically has a late onset, and is characterized by a post-BD forced expiratory flow rate in 1 s/forced vital capacity (FEV_1_/FVC) ratio < 0.70^7^. ACO is present in approximately 2.0% of the population worldwide and is associated with a very large healthcare burden^8,9^.

Lung function tests are physiologic markers of chronic airway disease, and traditional marker of COPD is FEV_1_^10^. As in COPD, annual FEV_1_ decline of ACO is more rapid than that of healthy adults^11–13^. When it compares to that of COPD or asthma, controversial results were found in lung function of ACO^14,15^. Moreover, their impact on clinical outcomes of ACO is controversial^16^.

There are not enough studies on which lung function indicators, such as FEV_1_ or FVC, are better for explanation of clinical outcomes in ACO. Interestingly, ACO patients showed significant difference in change of FVC, not that of FEV_1_^17^. In that study, they showed larger variation of FVC than that of FEV_1_^17^. They also showed different tendency of FVC changes compared to those of COPD^13^. FVC is an undervalued marker of lung function until these days. We hypothesized that FVC, not FEV_1,_ is associated with clinical outcomes of ACO. We conducted this study to compare FVC and FEV_1_ to predict the clinical outcomes of ACO, such as symptom scores, exercise capacity, and annual frequency and severity of exacerbations.

## Results

### Demographics according to FVC change

**Figure 1:**
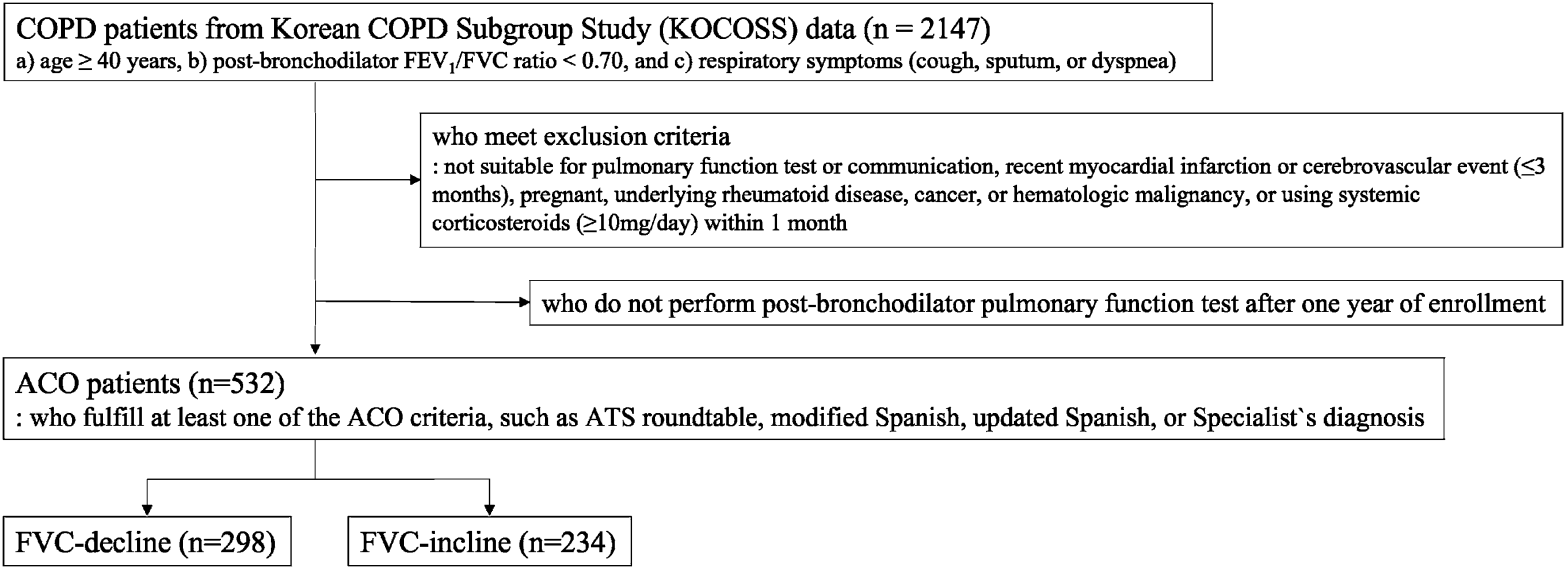
Flowchart of this study. Patients were extracted from KOCOSS study (n = 2147) who meet the COPD definition. If they satisfy the exclusion criteria or do not perform the pulmonary function tests 1 year after, they were excluded. Overall, 532 patients were finally enrolled in this study. They were divided into FVC-decline and FVC-incline group. Characteristics were compared between FVC-decline and FVC-incline. *ACO* asthma-COPD overlap, *ATS* American Thoracic Society, *COPD* chronic obstructive pulmonary disease, *FEV*_*1*_ forced expiratory volume in one second, *FVC* forced vital capacity.

The ACO criteria were fulfilled with 532 patients in the KOCOSS, of whom 298 were included in the FVC-decline group (Fig. 1). Age, sex, body mass index (BMI), or smoking status showed no statistical differences between the FVC-decline and FVC-incline groups. The proportion of patients with gastroesophageal reflux disease (GERD) was higher in the FVC-incline compared to FVC-decline group (21.4% vs. 14.1%, *p* = 0.03). However, the other comorbidities were not different between the groups. Baseline mean post-BD FVC values were higher in the FVC-decline group compared to the FVC-incline group (3.29 ± 0.76 vs. 2.99 ± 0.75 L, *p* < 0.01). The mean post-BD FEV_1_ values were not significantly different between the two groups. The annual change in FVC of the FVC-decline group was − 0.33 ± 0.29 L/year and that of the FVC-incline group was 0.26 ± 0.27 L/year. The annual change in FEV_1_ of the FVC-decline group was − 0.12 ± 0.21 L/year and that of the FVC-incline group was 0.16 ± 0.27 L/year. There was no statistical difference in medication use. Baseline St. George’s Respiratory Questionnaire-C (SGRQ-C) total scores were not significantly different between the groups. 6-min walk distance (6MWD) was lower in FVC-decline group than in FVC-incline group (378.5 m [320.0 {1st quartile}; 442.0 {3rd quartile}] vs. 401.5 m [328.0; 474.5], *p* < 0.05]. The history of past exacerbation within 1 year of study enrollment was not different between the groups (Table 1).

**Table 1:** Demographics according to FVC changes.

ACO patients	FVC-decline (n = 298)	FVC-incline (n = 234)	*p* value
Age (years), mean ± SD	69.1 ± 7.5	69.0 ± 7.5	0.93
Male sex, n (%)	276 (92.6)	220 (94.0)	0.52
BMI (kg/m^2)^, mean ± SD	23.2 ± 3.5	23.0 ± 3.5	0.50
**Smoking status, n (%)**			
Current smoker	68 (22.8)	63 (26.9)	0.22
Ex-smoker	209 (70.1)	148 (63.2)	
Never smoker	21 (7.1)	23 (9.8)	
Pack-year, packs ± SD	41.7 ± 29.5	38.7 ± 27.0	0.23
**Comorbidities, n (%)**			
Diabetes	52 (17.5)	49 (20.9)	0.32
Myocardial infarction	10 (3.4)	15 (6.4)	0.10
Congestive heart failure	12 (4.1)	10 (4.3)	0.90
Peripheral vascular disease	3 (1.0)	4 (1.7)	0.71
Hypertension	122 (41.2)	102 (43.6)	0.58
Osteoporosis	16 (5.4)	19 (8.2)	0.20
Gastro-esophageal reflux disease	42 (14.1)	50 (21.4)	0.03
**Lung function, mean ± SD**			
BD FVC (L)	3.29 ± 0.76	2.99 ± 0.75	< 0.01
BD FEV_1_ (L)	1.63 ± 0.57	1.54 ± 0.53	0.06
ΔFVC (L/year)	− 0.33 ± 0.29	0.26 ± 0.27	< 0.01
ΔFEV_1_ (L/year)	− 0.12 ± 0.21	0.16 ± 0.27	< 0.01
**Medication**			
None	31 (10.4)	27 (11.5)	0.65
Oral medication	12 (4.0)	18 (7.7)	
ICS	1 (0.3)	1 (0.4)	
LABA	19 (6.4)	12 (5.1)	
LAMA	57 (19.1)	43 (18.4)	
LABA/LAMA	38 (12.8)	27 (11.5)	
ICS/LABA	50 (16.8)	45 (19.2)	
ICA/LABA/LAMA	90 (30.2)	61 (26.1)	
**Baseline**			
SGRQ-C (score), median [1st quartile; 3rd quartile]			
Total	31.4 [19.3; 52.9]	29.2 [17.5; 48.3]	0.10
Symptom	35.5 [19.2; 50.7]	26.1 [14.5; 47.4]	0.01
Activity	42.7 [27.4; 59.8]	38.7 [25.3; 54.2]	0.08
Impact	20.7 [6.7; 41.8]	18.1 [5.6; 37.9]	0.17
6MWD (m), median [1st quartile; 3rd quartile]	378.5 [320.0; 442.0]	401.5 [328.0; 474.5]	< 0.05
≥ 350 m, n (%)	152 (61.3)	128 (68.8)	0.13
< 350 m, n (%)	96 (38.7)	58 (31.2)	
Exacerbation history (≤ 1 year), n (%)	57 (19.5)	56 (24.1)	0.23

Demographic data were summarized in Table 1. FVC-decline group showed lower underlying gastroesophageal reflux and higher baseline FVC compared to FVC-incline group. FVC-decline group have higher SGRQ-C symptom score and lower 6MWD than FVC-incline group.

*ACO* asthma-chronic obstructive pulmonary disease overlap, *BD* post-bronchodilator, *BMI* body mass index, *Δ* annual changes of the postbronchodilator values, *FEV*_*1*_ forced expiratory volume in one second, *FVC* changes of forced vital capacity, *SD* standard deviation, *SGRQ-C* St. George’s Respiratory Questionnaire-Chronic obstructive pulmonary disease specific version, *6MWD* 6-min walking distance.

### Clinical outcomes of ACO in 1 year are varied by FVC change

The SGRQ-C scores and the occurrence and frequency of acute exacerbations (AEs) during the 1-year follow-up were compared between the groups. One year after, SGRQ-C total scores were higher in FVC-decline group than in FVC-incline group (35.5 [19.2; 50.7] vs. 26.1 [14.5; 47.4], *p* = 0.01). In subgroup analyses, SGRQ-C symptom scores did not show the statistical difference between the FVC-decline and FVC-incline group. On the other hand, the SGRQ-C activity score (45.4 [29.8; 67.8] vs. 37.6 [22.5; 59.7], *p* = 0.01) and the SGRQ-C impact score (21.9 [5.1; 40.1] vs. 14.2 [4.6; 35.0], *p* = 0.03) were higher in the FVC-decline group than in the FVC-incline group. Patients in the FVC-decline group, compared to those in the FVC-incline group, experienced more moderate COPD exacerbation (47.1% vs. 36.8%, *p* = 0.02) and moderate-to-severe COPD exacerbation (49.8% vs. 39.6%, *p* = 0.03). An analysis of the number of exacerbations showed similar results. Moderate COPD exacerbations in the FVC-decline group were more frequent compared to the FVC-incline group (1.3 ± 2.1 times/year vs. 0.9 ± 1.7 times/year, *p* = 0.03). The number of moderate-to-severe COPD exacerbations was higher in the FVC-decline group compared to the FVC-incline group (1.5 ± 2.5 times/year vs. 1.1 ± 1.9 times/year, *p* = 0.04). There was no statistical difference in 6MWD and 1-year mortality between the two groups (Table 2).

**Table 2:** Difference of clinical outcomes in one year after according to the FVC grouping.

Clinical outcomes in 1 year after	FVC-decline (n = 298)	FVC-incline (n = 234)	*p* value
SGRQ-C (score), median [1st quartile; 3rd quartile]			
Total	35.5 [19.2; 50.7]	26.1 [14.5; 47.4]	0.01
Symptom	40.1 [26.3; 58.2]	38.1 [23.7; 54.6]	0.16
Activity	45.4 [29.8; 67.8]	37.6 [22.5; 59.7]	0.01
Impact	21.9 [5.1; 40.1]	14.2 [4.6; 35.0]	0.03
6MWD (m), median [1st quartile; 3rd quartile]	382.0 [300.0; 450.0]	401.5 [311.5; 441.0]	0.50
AEs frequency, times/year ± SD			
Moderate	1.3 ± 2.1	0.9 ± 1.7	0.03
Severe	0.2 ± 0.9	0.2 ± 0.7	0.59
Moderate-to-severe	1.5 ± 2.5	1.1 ± 1.9	0.04
AEs, n (%)			
Moderate	124 (47.1)	78 (36.8)	0.02
Severe	25 (9.5)	20 (9.4)	0.98
Moderate-to-severe	131 (49.8)	84 (39.6)	0.03
1-year mortality, n (%)	10 (3.4)	2 (0.9)	0.10

Clinical outcomes of one year after were compared between FVC-decline group and FVC-incline group. SGRQ-C total scores were significantly higher in FVC-decline group than in FVC-incline group. Those were similar in SGRQ-C activity and impact scores. Annual frequency of moderate and moderate-to-severe exacerbations were significantly higher in FVC-decline group than in FVC-incline group. Percentage of the patients who have exacerbation for one year showed similar results.

*AEs* acute exacerbations, *Δ* annual changes of the postbronchodilator values, *FVC* forced vital capacity, *SD* standard deviation, *SGRQ-C* St. George’s Respiratory Questionnaire-Chronic obstructive pulmonary disease specific version, *6MWD* 6-min walking distance.

### Comparison of SGRQ-C and acute exacerbations in 1 year after by ΔFVC quartile and ΔFEV_1_ quartile

**Figure 2:**
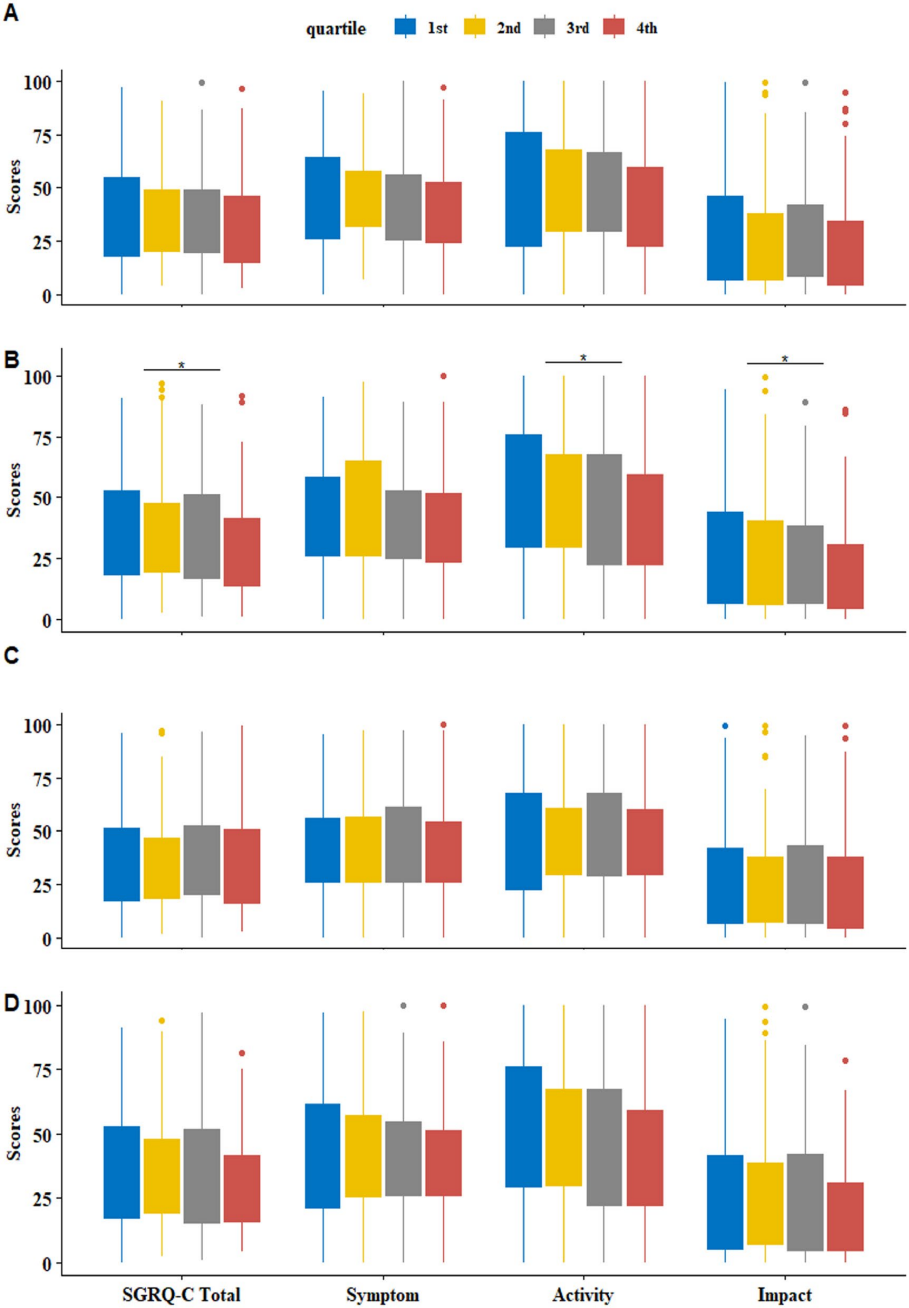
SGRQ-C in year after by ΔFVC quartile and ΔFEV_1_ quartile. For comparison, post-hoc analyses by Bonferroni method were performed between the groups, such as ΔFVC quartile and ΔFEV_1_ quartile. Baseline SGRQ-C by ΔFVC quartile (**A**) and ΔFEV_1_ quartile (**C**) did not show statistical difference between four groups in post-hoc analyses. Comparison of SGRQ-C (1 year) by ΔFVC quartile (**B**) showed a significant difference between the groups in post-hoc analyses. Especially, SGRQ-C activity and impact scores were different between the four groups and showed negative relationship according to the increase of ΔFVC quartile. On the other hand, SGRQ-C (1 year) by Δ FEV_1_ quartile (**D**) did not show the statistical difference between the groups. *p value < 0.05. *Δ* annual changes of the postbronchodilator values, *FEV*_*1*_ forced expiratory volume in one second, *FVC* forced vital capacity, *SGRQ-C* St. George’s Respiratory Questionnaire-Chronic obstructive pulmonary disease specific version.

FEV_1_-decline group also showed higher SGRQ-C total score, activity score, and impact scores than FEV_1_-incline group (Supplementary Table 1). Therefore, the association of ΔFVC (delta FVC: annual changes of the postbronchodilator values of FVC) quartile with SGRQ-C and that of ΔFEV_1_ quartile with SGRQ-C were evaluated for distinguishing better marker for SGRQ-C. Post-hoc analyses by Bonferroni method were performed between the groups. There was no statistical difference of baseline SGRQ-C by ΔFVC quartile (Fig. 2A) and ΔFEV_1_ quartile (Fig. 2C). It is also found in comparing the SGRQ-C in 1-year after by ΔFEV_1_ quartile (Fig. 2D). However, SGRQ-C (1 year) was improved as increasing order of ΔFVC quartile, especially in SGRQ-C total score, SGRQ-C activity score, and SGRQ-C impact scores (*p* < 0.05, each) (Fig. 2B). Therefore, FVC grouping explains respiratory related symptoms better than FEV_1_ grouping.

**Figure 3:**
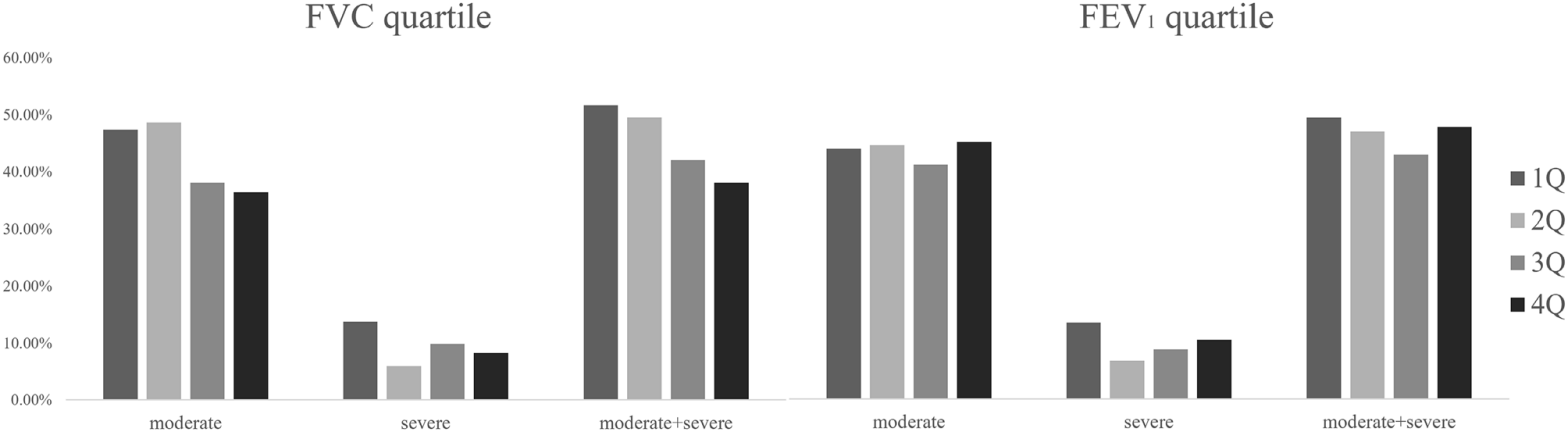
The percentage of patients who experienced COPD exacerbation in a year by ΔFVC quartile and ΔFEV_1_ quartile. FVC quartile showed the decreasing tendency of moderate-to-severe exacerbation without clinical significance. In the comparison by FEV_1_ quartiles, they did not show any significant differences. *COPD* chronic obstructive pulmonary disease, *Δ* annual changes of the postbronchodilator values, *FEV*_*1*_ forced expiratory volume in one second, *FVC* forced vital capacity, *Q* quartile.

Compared to those of FVC, the number and percentage of who experienced exacerbations were not different between the FEV_1_-decline and the FEV_1_-incline group (Supplementary Table 1). The scatter plots of ΔFVC and ΔFEV_1_ by annual frequency of COPD exacerbation (Supplementary Fig. 1) showed that trend lines and linear regression of ΔFVC correlated with annual AE frequency (*p* < 0.01), but ΔFEV_1_ did not (*p* = 0.77). In the comparing the association of both ΔFVC quartile and ΔFEV_1_ quartile with the percentage of AEs, they did not show statistically significant difference between the groups. However, ΔFVC quartile showed decreasing tendency in moderate COPD exacerbation and moderate-to-severe COPD exacerbation (Fig. 3).

### Differences between the exacerbation and non-exacerbation groups

We compared the clinical characteristics of patients who experienced and those who did not experience exacerbations. BMI was lower in the moderate AE group compared to non-AE group (22.7 ± 3.3 vs. 23.5 ± 3.6, *p* < 0.01). Other demographic data, such as age, sex, and smoking status, were not different between the two groups. Osteoporosis was more common in the moderate AE group compared to the non-AE group (10.9% vs. 4.1%, *p* < 0.01), while other comorbidities had a similar frequency between the groups. Osteoporosis was also more frequent in the moderate-to-severe AE group compared to the non-AE group (11.2% vs. 3.5%, *p* < 0.01). The history of past AEs was higher in AE group than in non-AE group (moderate AEs [32.5% vs. 16.2%, *p* < 0.01] and moderate-to-severe AEs [32.9% vs. 15.1%], both *p* < 0.01). The proportion of baseline 6MWD was different between the AE group and the non-AE group, but no significant differences were observed for other demographic data.

A higher percentage of FVC-decline patients were included in the moderate and moderate-to-severe AE groups. FVC-decline patients constituted 61.4% of the moderate AE group compared to 50.9% of the non-AE group (*p* = 0.02). Similarly, FVC-decline patients constituted 60.9% of the moderate-to-severe AE group compared to 50.8% of the non-AE group (*p* = 0.03). Conversely, the proportion of FEV_1_-decline patients was not different between the moderate and moderate-to-severe AE groups (*p* = 0.87 and *p* = 0.70, respectively) (Table 3).

**Table 3:** Clinical characteristics according to exacerbations in asthma-COPD overlap.

	Moderate AEs			Moderate-to-severe AEs		
	Yes (n = 202)	No (n = 273)	*p* value	Yes (n = 215)	No (n = 260)	*p* value
Age (years), mean ± SD	69.4 ± 7.0	69.0 ± 7.7	0.57	69.5 ± 7.1	68.8 ± 7.6	0.36
Male sex, n (%)	186 (92.1)	258 (94.5)	0.29	199 (92.6)	245 (94.2)	0.46
BMI (kg/m^2^), mean ± SD	22.7 ± 3.3	23.5 ± 3.6	< 0.01	22.8 ± 3.4	23.5 ± 3.5	0.05
**Smoking status, n (%)**						
Current smoker	49 (24.3)	63 (23.1)	0.20	52 (24.2)	60 (23.1)	0.33
Ex-smoker	141 (69.8)	181 (66.3)		149 (69.3)	173 (66.5)	
Never smoker	12 (5.9)	29 (10.6)		14 (6.5)	27 (10.4)	
Pack-year, mean ± SD	41.7 ± 26.7	38.6 ± 26.8	0.23	42.7 ± 28.0	37.6 ± 25.6	0.05
**Comorbidities, n (%)**						
Diabetes	35 (17.4)	57 (20.9)	0.35	39 (18.2)	53 (20.4)	0.55
MI	14 (7.0)	10 (3.7)	0.11	15 (7.0)	9 (3.5)	0.08
CHF	7 (3.5)	13 (4.8)	0.49	8 (3.7)	12 (4.6)	0.63
PVD	3 (1.5)	4 (1.5)	1.00	3 (1.4)	4 (1.6)	0.89
Hypertension	78 (38.6)	117 (43.2)	0.32	86 (40.0)	109 (42.2)	0.62
Osteoporosis	22 (10.9)	11 (4.1)	< 0.01	24 (11.2)	9 (3.5)	< 0.01
GERD	42 (20.8)	44 (16.1)	0.19	46 (21.4)	40 (15.4)	0.09
6MWD < 350 m, n (%)	85 (47.8)	58 (27.1)	< 0.01	88 (46.8)	55 (27.0)	< 0.01
Exacerbation history (≤ 1 year), n (%)	65 (32.5)	44 (16.2)	< 0.01	70 (32.9)	39 (15.1)	< 0.01
**Grouping, n (%)**						
FEV_1_-decline	107 (53.0)	142 (52.2)	0.87	115 (53.5)	134 (51.7)	0.70
FVC-decline	124 (61.4)	139 (50.9)	0.02	131 (60.9)	132 (50.8)	0.03

Clinical characteristics were compared between the two groups, such as the patients who experience the exacerbations or not. In the comparison of moderate AEs, BMI, underlying osteoporosis, and percentage of FVC-decline group were significantly different between the “Yes” group and “No” group. In the comparison of moderate-to-severe AEs, underlying osteoporosis and percentage of FVC-decline group were significantly different between the “Yes” group and “No” group.

*AEs* acute exacerbations, *BMI* body mass index, *CHF* congestive heart failure, *COPD* chronic obstructive pulmonary disease, *FEV*_*1*_ forced expiratory volume in one second, *FVC* forced vital capacity, *GERD* gastro-esophageal reflux disease, *MI* myocardial infarction, *PVD* peripheral vascular disease, *6MWD* 6-min walking distance, *SD* standard deviation.

### FVC-decline group, not FEV_1_-decline group, was associated with exacerbation in ACO patients

Univariable and multivariable logistic regression analyses were used to evaluate the factors associated with exacerbations in ACO patients (Table 4). Analyses were performed by two models, such as model 1 for ΔFVC and model 2 for ΔFEV_1_. Age, sex, BMI, 6MWD, and history of past AEs were selected as common variables because these were associated with next AEs in many previous articles^10,18^. Osteoporosis, and GERD were selected as variables because these were different factors in demographic analyses (FVC-decline vs. FVC-incline group and AEs “Yes” vs. AEs “No” group). In the unadjusted analyses, FVC-decline group (odds ratio [OR] = 1.53; 95% confidence interval [CI] 1.06–2.22; *p* = 0.02) was associated with moderate COPD exacerbation. After adjusted, FVC-decline group (OR = 1.58; 95% CI 1.02–2.44; *p* = 0.04) was still associated with moderate COPD exacerbation. For moderate-to-severe AEs, FVC-decline group showed significant association both in unadjusted analysis (OR 1.51; 95% CI 1.05–2.18, *p* = 0.03) and in adjusted analysis (OR 1.56; 95% CI 1.01–2.41, *p* < 0.05). On the other hand, FEV_1_-decline group did not showed association with moderate and moderate-to-severe COPD exacerbation both in unadjusted and in adjusted analyses. Details for other variables were summarized in Supplementary Table 3.

**Table 4:** Factors associated with exacerbation of ACO patients in univariable and multivariable logistic regression analysis.

	Moderate AEs				Moderate-to-severe AEs			
	Unadjusted		Adjusted*		Unadjusted		Adjusted*	
	OR (95% CI)	*p* value	OR (95% CI)	*p* value	OR (95% CI)	*p* value	OR (95% CI)	*p* value
Model 1 FVC-decline	1.53 (1.06–2.22)	0.02	1.58 (1.02–2.44)	0.04	1.51 (1.05–2.18)	0.03	1.56 (1.01–2.41)	< 0.05
Model 2 FEV_1_-decline	1.03 (0.72–1.49)	0.87	1.09 (0.71–1.68)	0.69	1.07 (0.75–1.54)	0.71	1.15 (0.75–1.77)	0.52

Univariable and multivariable logistic regression analyses of moderate AEs and moderate-to-severe AEs were performed. In the model 1, variables were selected from different factors of previous analyses (FVC-decline group vs. FVC-incline group and AEs “Yes” group vs. AEs “No” group). Compared to the FVC grouping of model 1, FEV_1_ grouping (FEV_1_-decline and FEV_1_ incline group) was selected as variables in model 2. After adjusted, FVC-decline group of model 1 was associated with moderate (OR 1.65) and moderate-to-severe AEs (OR 1.65), which was not shown in FEV_1_ grouping of model 2.

*ACO* asthma-chronic obstructive pulmonary disease overlap, *AEs* acute exacerbations, *BMI* body mass index, *CI* confidence interval, *FEV*_*1*_ forced expiratory volume in one second, *FVC* forced vital capacity, *GERD* gastro-esophageal reflux disease, *6MWD* 6-min walking distance, *OR* odds ratio.

*Adjusted by age, sex, BMI, osteoporosis, GERD, 6MWD, and history of AEs within 1 year.

## Discussion

Traditional lung function test variables, such as FEV_1_, are associated with long-term mortality in COPD patients^19^. However, they are not strongly associated with the respiratory symptoms, quality of life, or exacerbation of these patients^20,21^. Therefore, Global Initiative for Chronic Obstructive Lung Disease guidelines recommend the management by the symptom and risk of exacerbation^10^. It is important but frequently overlooked that COPD is diagnosed based on the FEV_1_/FVC ratio, not FEV_1_ alone. However, the role of FVC in COPD evaluation is not clear. Previous studies have reported that the variation in FVC among COPD patients, particularly before and after an exacerbation, was greater compared to that of FEV_1_^17,22^. A similar situation was observed for ACO patients. Large longitudinal study showed that FVC changes in ACO was different from that of healthy adults, like in COPD. However, no previous studies have focused on FVC changes as prognostic value for clinical outcomes in ACO^13^.

In this ACO study, we identified some interesting points of FVC changes. First, grouping by FVC changes well explained symptoms and exacerbation of ACO after 1 year. The FVC-decline group existed in 56% of ACO patients. The SGRQ-C scores in 1 year after were higher in the FVC-decline group than in the FVC-incline group, especially in total, activity, and impact scores. They also showed negative relationship in comparison by quartile range. The annual frequency of COPD exacerbation was higher in FVC-decline group than in FVC-incline group. After adjusting confounding variables, the risk of moderate (OR 1.58) and moderate-to-severe exacerbation (OR 1.56) was higher in the FVC-decline group than in the FVC-incline group. These results supported the value of FVC changes in ACO explanation, which was not seen in previous studies. Theoretical mechanism of these results can be explained by increased residual volume (RV) or air-trapping. There were many reports that RV/total lung capacity (TLC) was the markers of lung hyperinflation and were associated with AEs^23–25^. FVC-decline is coincided with increased RV, which finally leads to increased RV/TLC^13,17^. In a recent study, Alter et al. showed that FVC was an alternative value of RV/TLC, which represented air-trapping or pulmonary hyperinflation^26^. Especially in absence of body plethysmography, FVC can be a good lung function marker on behalf of the role of RV/TLC as a predictor of COPD exacerbations. Further studies should be followed including subjects such as the cut-off values of FVC changes for predicting next AEs or possibility of FVC changes as COPD lung function phenotypes in near future.

Second, FVC changes were valuable than FEV_1_ changes in explaining exacerbation of ACO. In the logistic regression analyses, FVC changes had prognostic value which was not shown in FEV_1_ changes. We also showed the decreasing trends of moderate and moderate-to-severe exacerbations as the quartile of FVC changes increases. These highlighted the importance of FVC (but not FEV_1_) which was associated with exacerbation in ACO. ACO showed higher BDR of FEV_1_ which means that FEV_1_ can be affected by other conditions such as individual efforts, medication, and exacerbation^17^. It is the possible reason why FVC changes showed significant results compared to FEV_1_ changes.

There were several limitations in this study. First, we did not compare the outcomes between different ACO diagnostic criteria. However, the diagnosis criteria of ACO have not been fully established. We did not want to miss any cases of ACO to avoid the selection bias. Future studies should compare outcomes between different ACO diagnostic criteria. Second, we only used the 1-year follow-up data. However, we set this study to understand the intuitive relationship between FVC changes and outcomes of ACO management. We showed the clinical difference of SGRQ-C changes by FVC grouping compared to those of by FEV_1_ grouping. Also, we showed the association of exacerbation with FVC changes as originally hypothesized. Further study should be analyzed in details for evaluating consistent results by the long-term follow-up data. Third, there was no contained radiographic data for analyzing the relationship between FVC changes and air trapping in this study. However, we previously described that FVC itself was a good marker of air trapping. Future study about the association of the diameter of bronchi or low attenuation area that are measured by chest tomography scan with FVC changes should be good subjects to evaluate.

This study is the first to report the importance of FVC changes that it is associated with exacerbations in ACO. FVC changes should be considered in managing ACO patients.

## Methods

### Data sources and protocols

The Korea COPD Subgroup Study (KOCOSS) cohort is a prospective, multicenter, and observational cohort study that recruited participants from 58 referral university hospitals in South Korea. The KOCOSS protocol was registered at www.clinicaltrial.gov (NCT02800499). For the current study, we used 1-year follow-up data from the KOCOSS database. All participants were recruited between 2012 and 2019. Details of the KOCOSS cohort have been described previously^27–29^.

### Study population and design

KOCOSS cohort included COPD patients aged ≥ 40 years with a post-BD FEV_1_/FVC ratio < 0.70, and respiratory symptoms such as cough, sputum production, or dyspnea^29^. Patients were excluded if they are (1) not suitable for pulmonary function test or communication, (2) recent myocardial infarction or cerebrovascular event (≤ 3 months), (3) pregnant, (4) underlying rheumatoid disease, (5) cancer or hematologic malignancy patients, or (6) used systemic corticosteroid (≥ 10 mg/day) within 1 month. Among KOCOSS cohort participants, we extracted ACO patients who was diagnosed based on various diagnostic criteria, such as the modified Spanish, updated Spanish, and American Thoracic Society (ATS) roundtable criteria, as well as a specialist’s diagnosis^30,31^. Patients were included if they fulfill at least one of the ACO criteria.

1. Modified Spanish COPD guideline criteria (at least 1 major or 2 minor)
  (A) Major: (i) previous history of asthma, (ii) Bronchodilator response (BDR) > 15% and 400 mL
  (B) Minor: (i) Ig E > 100 IU, or history of atopy, (ii) BDR > 12% and 200 mL, (iii) blood eosinophil > 5%
2. Updated Spanish COPD guideline criteria (A and B or A and C)
  (A) Age ≥ 35, ≥ 10 pack-year smoking history, and post-BD FEV_1_/FVC < 0.7
  (B) Current diagnosis of asthma
  (C) BDR > 15% and 400 mL, and/or blood eosinophilia ≥ 300 cell/μL
3. ATS roundtable criteria
  (A) Major: (i) post-BD FEV_1_/FVC < 0.7 and Age ≥ 40, (ii) ≥ 10 pack-year smoking or exposure to air pollution ≥ 10 years, (iii) history of asthma before 40-year-old or BDR > 400 mL in FEV_1_
  (B) Minor: (i) history of atopy or allergic rhinitis, (ii) separate BDR ≥ 12% and 200 mL, (iii) blood eosinophil ≥ 300 cell/μL
4. Specialist’s diagnosis: answer yes to this question “Is this patient likely to be classified as ACO?”

We excluded patients who did not undergo the follow-up lung function tests. We divided the patients into FVC-decline and FVC-incline groups on the basis of post-BD FVC changes. We compared the demographics between the groups. To analyze the predictive value of FVC and FEV_1_, clinical outcomes such as respiratory symptoms and AEs were compared. We also compared the ability of FVC and FEV_1_ changes to predict exacerbations during the 1-year follow up.

### Study variables

The demographic data of patients were collected at KOCOSS enrollment. Age, sex, BMI, smoking status, and comorbidities such as myocardial infarction, heart failure, peripheral vessel disease, diabetes, hypertension, osteoporosis, and GERD were recorded. Pulmonary function tests (PFT, including pre- and post-BD tests) were conducted at baseline. Symptoms and exercise capacity were evaluated using the modified Medical Research Council scale, SGRQ-C, and 6MWD. History of AEs within one year was recorded also at the enrollment. At 1 year, all variables including PFT with post-BD tests, SGRQ-C, and 6MWD were gathered at the same time. All tests were voluntarily performed. If patients do not want, they did not perform the previously mentioned tests under free will. Moderate and severe AE were recorded also.

### Definition of exacerbation

Currently, there is no established consensus of AEs in ACO. Moreover, the definition of AEs in COPD differs from that in asthma. The definition of exacerbations in this study was adopted from the guideline of Global Initiative for Chronic Obstructive Lung Disease (GOLD), because all cohort data was originally targeted COPD patients^10^. As previously mentioned in GOLD guideline, AE was defined by acute change of respiratory symptoms, which need medication changes such as systemic corticosteroid or antibiotics^10^. Among them, severe AE was defined who requires hospitalization or care of emergency room for management of changed symptoms^32^.

### Statistical analyses

We used the Student’s *t*-test for compare continuous variables. Pearson’s chi-square test and Fisher’s exact test were used to compare categorical variables. Kruskal–Wallis test was used for comparing the means of multiple groups and Bonferroni method was used for post-hoc analyses. Univariable and multivariable logistic regression analyses were used to identify predictors of AE. *P* values < 0.05 were considered statistically significance. The statistical analyses were performed using RStudio (version: 2020; RStudio, Inc., Boston, MA, USA).

### Ethical approval and consent to participate

Current study was conducted according to the Helsinki declaration, and it was approved by the Ethics Committee of each participating medical center. Relevant data was provided by anonymous form. All participants provided written informed consent prior to enrollment. The lists of participated medical centers and their Ethics Committee were as follows: Gachon University Gil Medical Center, Gangnam Severance Hospital, Gangdong Kyung Hee University Hospital, Kangbuk Samsung Hospital, Gangwon National University Hospital, Konkuk University Medical Center, Kyungpook National University Hospital, Kyung Hee University Medical Center, Keimyung University Dongsan Medical Center, Korea University Guro Hospital, Korea University Anam Hospital, Dong-A University Hospital, Pusan National University Hospital, The Catholic University of Korea Bucheon St. Mary’s Hospital, Soonchunhyang University Hospital, Bundang Cha Medical Center, Nowon Eulji Medical Center, Seoul National University Hospital, Seoul Metropolitan Government-Seoul National University Boramae Medical Center, Samsung Medical Center, The Catholic University of Korea Seoul St. Mary’s Hospital, The Catholic University of Korea St. Vincent’s Hospital, Severance Hospital, Ajou University Hospital, The Catholic University of Korea Yeouido St. Mary’s Hospital, Yeungnam University Medical Center, Yongin Severance Hospital, Ulsan University Hospital, Dongkang Hopital, wonkwang university Hospital, Wonju Severance Christian Hospital, The Catholic University of Korea, Eunpyeong St. Mary's Hospital, The Catholic University of Korea Uijeongbu St. Mary’s Hospital, Ewha Womans University Medical Center, The Catholic University of Korea Incheon St. Mary’s Hospital, Inje University Ilsan Paik Hospital, Chosun University Hospital, Chonnam National University Hospital, Jeonbuk National University Hospital, Jeju National University Hospital, Gyeongsang National University Changwon Hospital, Chungnam National University Hospital, Hallym University Medical Center, Kangdong Sacred Heart Hospital, Hallym University Sacred Heart Hospital, Hanyang University Medical Center, Inje University Haeundae Paik Hospital.

## Supplementary Information

Supplementary Information.

## Data Availability

Researchers may send reasonable requests for access to the datasets used in this study to the corresponding author.

## References

[1] An TJ, Rhee CK, Kim JH, Lee YR, Chon JY, Park CK (2018). Effects of macrolide and corticosteroid in neutrophilic asthma mouse model. Tuberc. Respir. Dis. (Seoul).

[2] Viegi G, Pistelli F, Sherrill DL, Maio S, Baldacci S, Carrozzi L (2007). Definition, epidemiology and natural history of COPD. Eur. Respir. J..

[3] An TJ, Kim Y, Park YB, Kim K, Cho DY, Yoo KH (2021). Inhaled corticosteroid is not associated with a poor prognosis in COVID-19. Respirology.

[4] Barrecheguren M, Pinto L, Mostafavi-Pour-Manshadi SM, Tan WC, Li PZ, Aaron SD (2020). Identification and definition of asthma-COPD overlap: The CanCOLD study. Respirology.

[5] Pérez-de-Llano L, Cosio BG, Iglesias A, de las Cuevas N, Soler-Cataluña JJ, Izquierdo JL (2017). Asthma-COPD overlap is not a homogeneous disorder: Further supporting data. Respir. Res..

[6] Sin DD, Miravitlles M, Mannino DM, Soriano JB, Price D, Celli BR (2016). What is asthma−COPD overlap syndrome? Towards a consensus definition from a round table discussion. Eur. Respir. J..

[7] Yanagisawa S, Ichinose M (2018). Definition and diagnosis of asthma–COPD overlap (ACO). Allergol. Int..

[8] Hiles SA, Gibson PG, McDonald VM (2021). Disease burden of eosinophilic airway disease: Comparing severe asthma, COPD and asthma–COPD overlap. Respirology.

[9] Hosseini M, Almasi-Hashiani A, Sepidarkish M, Maroufizadeh S (2019). Global prevalence of asthma-COPD overlap (ACO) in the general population: A systematic review and meta-analysis. Respir. Res.h..

[10] 2020 Global Initiative for Chronic Obstructive Lung Disease I. Global Strategy for the diagnosis, management, and prevention of chronic obstructive pulmonary disease. Available from: https://goldcopd.org/2021-gold-reports/ (2021)

[11] Kim TB, Park CS, Bae YJ, Cho YS, Moon HB, Group CS (2009). Factors associated with severity and exacerbation of asthma: A baseline analysis of the cohort for reality and evolution of adult asthma in Korea (COREA). Ann. Allergy Asthma Immunol..

[12] Leem AY, Park B, Kim YS, Chang J, Won S, Jung JY (2019). Longitudinal decline in lung function: A community-based cohort study in Korea. Sci Rep..

[13] de Marco R, Marcon A, Rossi A, Antó JM, Cerveri I, Gislason T (2015). Asthma, COPD and overlap syndrome: A longitudinal study in young European adults. Eur. Respir. J..

[14] Park SY, Jung H, Kim JH, Seo B, Kwon OY, Choi S (2019). Longitudinal analysis to better characterize Asthma-COPD overlap syndrome: Findings from an adult asthma cohort in Korea (COREA). Clin. Exp. Allergy.

[15] Park HY, Lee S-Y, Kang D, Cho J, Lee H, Lim SY (2018). Favorable longitudinal change of lung function in patients with asthma-COPD overlap from a COPD cohort. Respir. Res..

[16] Jo YS (2021). Current status of studies investigating asthma-chronic obstructive pulmonary disease overlap in Korea: A review. Tuberc. Respir. Dis..

[17] Lim JU, Kim DK, Lee MG, Hwang YI, Shin KC, In KH (2020). Clinical characteristics and changes of clinical features in patients with asthma-COPD overlap in Korea according to different diagnostic criteria. Tuberc. Respir. Dis. (Seoul).

[18] Celli BR, Cote CG, Marin JM, Casanova C, Montes de Oca M, Mendez RA (2004). The body-mass index, airflow obstruction, dyspnea, and exercise capacity index in chronic obstructive pulmonary disease. N. Engl. J. Med..

[19] Menezes AMB, Pérez-Padilla R, Wehrmeister FC, Lopez-Varela MV, Muiño A, Valdivia G (2014). FEV1 is a better predictor of mortality than FVC: The PLATINO cohort study. PLoS ONE.

[20] Han MK, Muellerova H, Curran-Everett D, Dransfield MT, Washko GR, Regan EA (2013). GOLD 2011 disease severity classification in COPDGene: A prospective cohort study. Lancet Respir. Med..

[21] Jones PW (2009). Health status and the spiral of decline. COPD.

[22] Halpin DMG, Decramer M, Celli BR, Mueller A, Metzdorf N, Tashkin DP (2017). Effect of a single exacerbation on decline in lung function in COPD. Respir. Med..

[23] Shin TR, Oh YM, Park JH, Lee KS, Oh S, Kang DR (2015). The prognostic value of residual volume/total lung capacity in patients with chronic obstructive pulmonary disease. J. Korean Med. Sci..

[24] Kakavas S, Kotsiou OS, Perlikos F, Mermiri M, Mavrovounis G, Gourgoulianis K (2021). Pulmonary function testing in COPD: Looking beyond the curtain of FEV1. NPJ Primary Care Respir. Med..

[25] Kim Y, Kim SH, Rhee CK, Lee JS, Lee CY, Kim DK (2022). Air trapping and the risk of COPD exacerbation: Analysis from prospective KOCOSS cohort. Front. Med. (Lausanne)..

[26] Alter P, Orszag J, Kellerer C, Kahnert K, Speicher T, Watz H (2020). Prediction of air trapping or pulmonary hyperinflation by forced spirometry in COPD patients: Results from COSYCONET. ERJ Open Res..

[27] Choi JY, Kim SY, Lee JH, Park YB, Kim YH, Um SJ (2020). Clinical characteristics of chronic obstructive pulmonary disease in female patients: Findings from a KOCOSS cohort. Int. J. Chronic Obstruct. Pulm. Dis..

[28] Jo YS, Choe J, Shin SH, Koo HK, Lee WY, Kim YI (2020). Exhaled nitric oxide in patients with stable chronic obstructive pulmonary disease: Clinical implications of the use of inhaled corticosteroids. Tuberc. Respir. Dis. (Seoul).

[29] Lee JY, Chon GR, Rhee CK, Kim DK, Yoon HK, Lee JH (2016). Characteristics of patients with chronic obstructive pulmonary disease at the first visit to a pulmonary medical center in Korea: The Korea COPD subgroup study team cohort. JKMS.

[30] Jo YS, Hwang YI, Yoo KH, Kim TH, Lee MG, Lee SH (2019). Comparing the different diagnostic criteria of asthma-COPD overlap. Allergy.

[31] Jo YS, Lee J, Yoon HI, Kim DK, Yoo CG, Lee CH (2017). Different prevalence and clinical characteristics of asthma-chronic obstructive pulmonary disease overlap syndrome according to accepted criteria. Ann. Allergy Asthma Immunol..

[32] An TJY, Yoo YJ, Lim JU, Seo W, Park CK, Rhee CK, Yoon HK (2022). Diaphragm ultrasound is an imaging biomarker that distinguishes exacerbation status from stable chronic obstructive pulmonary disease. Int. J. Chronic Obstruct. Pulm. Dis..

