# Supplementary material for: FVC, but not FEV1, is associated with clinical outcomes of asthma-COPD overlap
Source: Sci Rep. 2022 Aug 15;12:13820. doi: 10.1038/s41598-022-15612-w (PMC9378661; doi:10.1038/s41598-022-15612-w)
Supplement: Supplementary file 1 — Supplementary Information. [file 41598_2022_15612_MOESM1_ESM.docx]

Supplementary Table 1. Demographics according to FEV_1_ changes

| ACO patients | FEV_1_-decline | FEV_1_-incline | *p*-value |
| --- | --- | --- | --- |
| SGRQ-C score (mean ± SD) |  |  |  |
| Total (baseline) | 30.8 [18.0;49.9] | 29.4 [18.9;50.9] | 0.54 |
| Symptom (baseline) | 39.1 [26.3;57.2] | 42.9 [26.3;58.9] | 0.60 |
| Activity (baseline) | 44.9 [29.5;67.6] | 44.9 [29.5;60.6] | 0.46 |
| Impact (baseline) | 21.2 [7.2;40.3] | 17.4 [5.0;39.8] | 0.21 |
| Total (1 year after) | 34.3 [18.5;51.2] | 26.5 [16.0;46.5] | 0.04 |
| Symptom (1 year after) | 39.2 [23.9;58.3] | 39.3 [26.3;52.7] | 0.77 |
| Activity (1 year after) | 44.9 [29.8;75.8] | 37.6 [22.5;59.7] | 0.02 |
| Impact (1 year after) | 22.0 [6.7;40.1] | 13.8 [4.6;35.0] | 0.03 |
| AEs frequency, times/year ± SD |  |  |  |
| Moderate | 1.1 ± 2.0 | 1.1 ± 1.9 | 0.98 |
| Severe | 0.2 ± 0.9 | 0.1 ± 0.6 | 0.40 |
| Moderate-to-Severe | 1.3 ± 2.4 | 1.3 ± 2.2 | 0.80 |
| AEs, n (%) |  |  |  |
| Moderate | 107 (43.0) | 95 (42.2) | 0.94 |
| Severe | 24 (9.6) | 21 (9.3) | 1.00 |
| Moderate-to-Severe | 115 (46.2) | 100 (44.4) | 0.77 |

AEs; acute exacerbations; ACO, asthma-chronic obstructive pulmonary disease overlap; FEV_1_, forced expiratory volume in one second; SGRQ-C, St. George`s Respiratory Questionnaire-COPD specific version; SD, standard deviation

Supplementary Table 2. Interquartile differences of SGRQ-C and acute exacerbations by FVC quartiles and by FEV_1_ quartiles

| ΔFVC quartile | | | | | |
| --- | --- | --- | --- | --- | --- |
|  | 1Q  (< -290) | 2Q  (-290 ~ -50) | 3Q  (-50 ~ 150) | 4Q  (≤ 150) | *p*-value |
| SGRQ-C score (mean ± SD) |  |  |  |  |  |
| Total (baseline) | 35.8 [18.1;54.7] | 28.6 [20.5;48.9] | 31.9 [19.5;49.3] | 27.4 [15.1;46.1] | 0.10 |
| Symptom (baseline) | 41.5 [26.3;64.3] | 43.6 [32.2;58.3] | 35.9 [25.6;56.4] | 38.9 [24.3;52.7] | 0.15 |
| Activity (baseline) | 53.1 [22.6;75.8] | 44.9 [29.8;67.6] | 44.9 [29.8;66.4] | 37.6 [22.5;59.3] | 0.07 |
| Impact (baseline) | 21.7 [6.8;46.2] | 18.5 [6.7;38.2] | 21.6 [8.6;42.0] | 15.2 [4.6;34.7] | 0.17 |
| Total (1 year after) | 37.8 [18.4;53.1] | 31.3 [19.5;47.8] | 30.3 [17.1;51.2] | 23.2 [13.8;41.3] | 0.01 |
| Symptom (1 year after) | 40.8 [26.0;58.3] | 39.8 [26.0;65.2] | 39.5 [24.9;52.7] | 34.2 [23.4;51.5] | 0.23 |
| Activity (1 year after) | 52.0 [29.6;76.0] | 44.9 [29.8;67.6] | 48.1 [22.5;67.6] | 37.1 [22.5;59.3] | 0.01 |
| Impact (1 year after) | 23.1 [6.7;44.5] | 16.9 [5.6;40.4] | 20.6 [6.0;38.1] | 12.2 [4.6;30.6] | 0.01 |
| Acute exacerbations (%) |  |  |  |  |  |
| Moderate | 55 (47.4) | 57 (48.7) | 46 (38.0) | 44 (36.4) | 0.12 |
| Severe | 16 (13.8) | 7 (6.0) | 12 (9.9) | 10 (8.3) | 0.22 |
| Moderate-to-Severe | 60 (51.7) | 58 (49.6) | 51 (42.1) | 46 (38.0) | 0.12 |
| ΔFEV_1_ quartile | | | | | |
|  | 1Q  (< -140) | 2Q  (-140 ~ -10) | 3Q  (-10 ~ 120) | 4Q  (≤ 120) | *p*-value |
| SGRQ-C (mean ± SD) |  |  |  |  |  |
| Total (baseline) | 29.0 [17.5;51.2] | 30.2 [18.7;47.0] | 28.2 [20.2;52.3] | 30.7 [16.1;50.8] | 0.93 |
| Symptom (baseline) | 37.7 [26.3;55.9] | 39.0 [26.0;56.4] | 44.4 [26.5;60.7] | 41.6 [26.0;54.9] | 0.76 |
| Activity (baseline) | 44.9 [22.5;67.6] | 44.9 [29.8;60.6] | 44.9 [29.5;67.6] | 44.9 [29.5;60.4] | 0.94 |
| Impact (baseline) | 21.2 [6.8;41.9] | 19.0 [7.6;37.9] | 17.1 [6.7;43.0] | 19.8 [4.6;37.8] | 0.83 |
| Total (1 year after) | 36.1 [17.4;52.8] | 32.0 [19.3;48.1] | 29.5 [15.3;51.9] | 24.8 [16.1;42.1] | 0.15 |
| Symptom (1 year after) | 39.3 [20.7;61.9] | 39.0 [25.6;56.8] | 39.3 [25.0;54.9] | 38.9 [26.3;51.3] | 0.97 |
| Activity (1 year after) | 47.0 [29.5;76.0] | 44.9 [29.8;67.6] | 41.8 [22.5;67.6] | 37.6 [22.5;59.3] | 0.21 |
| Impact (1 year after) | 22.0 [5.1;42.4] | 21.2 [6.7;38.7] | 18.7 [4.6;42.0] | 12.3 [4.8;31.0] | 0.13 |
| Acute exacerbations (%) |  |  |  |  |  |
| Moderate | 50 (43.5) | 53 (43.1) | 47 (39.8) | 52 (43.7) | 0.924 |
| Severe | 15 (13.0) | 8 (6.5) | 10 (8.5) | 12 (10.1) | 0.367 |
| Moderate-to-Severe | 55 (47.8) | 56 (45.5) | 49 (41.5) | 55 (46.2) | 0.797 |

ΔFEV_1_, changes of forced expiratory volume in 1 second; ΔFVC, changes of forced vital capacity; SD, standard deviation; SGRQ-C, St. George`s Respiratory Questionnaire-COPD specific version; Q, quartile

Supplementary Table 3. Detailed results of univariable and multivariable logistic regression analyses by different models (FVC and FEV_1_)

|  | Moderate AEs | | | | Moderate-to-Severe AEs | | | |
| --- | --- | --- | --- | --- | --- | --- | --- | --- |
|  | Univariable | | Multivariable | | Univariable | | Multivariable | |
| Model 1 | OR (95% CI) | *p*-value | OR (95% CI) | *p*-value | OR (95% CI) | *p*-value | OR (95% CI) | *p*-value |
| Age (year) | 1.01 (0.98-1.03) | 0.57 | 0.99 (0.96-1.02) | 0.45 | 1.01 (0.99-1.04) | 0.35 | 0.99 (0.96-1.02) | 0.64 |
| Male sex | 0.68 (0.33-1.40) | 0.29 | 0.62 (0.27-1.47) | 0.28 | 0.76 (0.37-1.58) | 0.46 | 0.74 (0.31-1.75) | 0.49 |
| BMI (kg/m^2^) | 0.93 (0.88-0.98) | 0.01 | 0.93 (0.87-0.99) | 0.02 | 0.95 (0.90-1.00) | 0.06 | 0.93 (0.88-0.99) | 0.03 |
| Osteoporosis | 2.89 (1.37-6.11) | < 0.01 | 2.79 (1.14-6.83) | 0.03 | 3.48 (1.58-7.65) | < 0.01 | 3.82 (1.44-10.14) | < 0.01 |
| GERD | 1.37 (0.86-2.18) | 0.19 | 1.20 (0.68-2.11) | 0.52 | 1.50 (0.94-2.39) | 0.09 | 1.31 (0.74-2.31) | 0.35 |
| 6MWD < 350m | 2.46 (1.61-3.75) | < 0.01 | 2.31 (1.45-3.67) | < 0.01 | 2.38 (1.56-3.63) | < 0.01 | 2.19 (1.38-3.49) | < 0.01 |
| AEs (≤ 1 year) | 2.49 (1.61-3.87) | < 0.01 | 2.44 (1.45-4.12) | < 0.01 | 2.76 (1.77-4.31) | < 0.01 | 2.56 (1.50-4.36) | < 0.01 |
| FVC-decline | 1.53 (1.06-2.22) | 0.02 | 1.58 (1.02-2.44) | 0.04 | 1.51 (1.05-2.18) | 0.03 | 1.56 (1.01-2.41) | < 0.05 |
| Model 2 |  |  |  |  |  |  |  |  |
| Age (year) | 1.01 (0.98-1.03) | 0.57 | 0.99 (0.96-1.02) | 0.40 | 1.01 (0.99-1.04) | 0.35 | 0.99 (0.96-1.02) | 0.59 |
| Male sex | 0.68 (0.33-1.40) | 0.29 | 0.59 (0.25-1.38) | 0.22 | 0.76 (0.37-1.58) | 0.46 | 0.70 (0.30-1.65) | 0.41 |
| BMI (kg/m^2^) | 0.93 (0.88-0.98) | 0.01 | 0.93 (0.87-0.99) | 0.03 | 0.95 (0.90-1.00) | 0.06 | 0.94 (0.88-1.00) | 0.04 |
| Osteoporosis | 2.89 (1.37-6.11) | < 0.01 | 2.68 (1.09-6.57) | 0.03 | 3.48 (1.58-7.65) | < 0.01 | 3.71 (1.40-9.85) | < 0.01 |
| GERD | 1.37 (0.86-2.18) | 0.19 | 1.16 (0.67-2.03) | 0.60 | 1.50 (0.94-2.39) | 0.09 | 1.28 (0.73-2.24) | 0.40 |
| 6MWD < 350m | 2.46 (1.61-3.75) | < 0.01 | 2.36 (1.49-3.74) | < 0.01 | 2.38 (1.56-3.63) | < 0.01 | 2.24 (1.41-3.55) | < 0.01 |
| AEs (≤ 1 year) | 2.49 (1.61-3.87) | < 0.01 | 2.35 (1.40-3.94) | < 0.01 | 2.76 (1.77-4.31) | < 0.01 | 2.48 (1.46-4.20) | < 0.01 |
| FEV_1_-decline | 1.03 (0.72-1.49) | 0.87 | 1.09 (0.71-1.68) | 0.69 | 1.07 (0.75-1.54) | 0.71 | 1.15 (0.75-1.77) | 0.52 |

AEs; acute exacerbations; BMI, body mass index; CI, confidence interval; FEV_1_, forced expiratory volume in one second; FVC, forced vital capacity; GERD, gastro-esophageal reflux disease; OR, odds ratio

Supplementary Figure 1. Trends lines of FVC and FEV_1_ with annual frequency of exacerbations


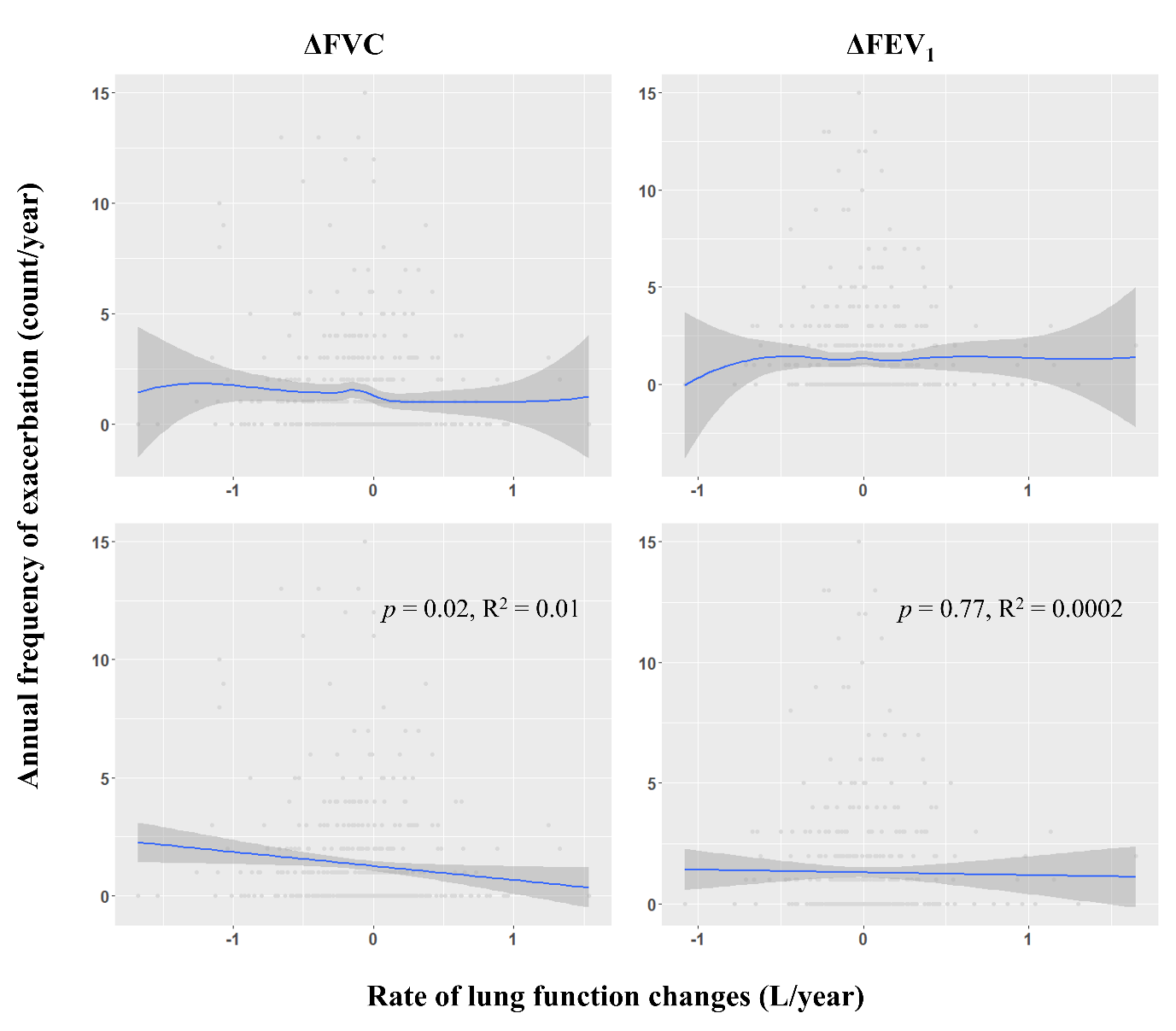


Trends lines of scatter plots of changes of FVC and forced expiratory volume in 1 second (FEV_1_) is calculated by linear regression analysis. Δ, annual changes of the postbronchodilator values; FEV_1_, forced expiratory volume in one second; FVC, forced vital capacity
